# Factors Associated With In-Hospital Death Among Pneumonia Patients in US Hospitals From 2016~2019

**DOI:** 10.34172/ijhpm.2023.7390

**Published:** 2023-07-24

**Authors:** Sun Jung Kim, Mar Medina, Lixian Zhong, Jongwha Chang

**Affiliations:** ^1^Department of Health Administration and Management, College of Medical Science, Soonchunhyang University, Asan, Republic of Korea; ^2^Center for Healthcare Management Science, Soonchunhyang University, Asan, Republic of Korea; ^3^Department of Software Convergence, Soonchunhyang University, Asan, Republic of Korea; ^4^School of Pharmacy, University of Texas at El Paso, El Paso, TX, USA; ^5^Department of Pharmaceutical Sciences, Irma Lerma Rangel School of Pharmacy, Texas A&M University, College Station, TX, USA

**Keywords:** Pneumonia, NIS Sample, In-Hospital Death, Health Disparity

## Abstract

**Background:** Pneumonia is one of the leading causes of hospital admission in the United States with a global health burden of about 6.8 million hospitalizations and 1.1 million deaths in patients over 65 years old in 2015. This study aimed to identify possible patient and hospital-related risk factors for in-hospital pneumonia death across US hospitals.

**Methods:** The National Inpatient Sample (NIS) was used to identify nationwide pneumonia patients (n=374 766, weighted n=1 873 828) from 2016 to 2019. We examined the characteristics of the study sample and their association with in-hospital death. Multivariate survey logistic regression models were used to identify risk factors.

**Results:** During the study periods, in-hospital death rates continuously decreased (2.45% in 2016 to 2.19% in 2019). Descriptive statistics showed that patient and hospital factors had varied in-hospital death rates. Survey logistic regression results suggested that male, very low income, non-Medicare, government hospitals, rural hospitals, and specific hospital regions were associated with higher in-hospital death rates than their reference groups.

**Conclusion:** Socioeconomic factors, including income and insurance, are associated with pneumonia mortality. Census region, hospital ownership, and rural location are also related to in-hospital mortality. Such findings in underserved, impoverished, and rural areas to identify possible health disparities.

## Background

**Key Messages**

**Implications for policy makers**

- Pneumonia is one of the leading causes of hospital admission in the United States.
- It is necessary to understand patient and hospital factors that increase pneumonia mortality rates outside of common clinical characteristics.
- Socioeconomic status and race have important implications on pneumonia outcomes.
- This study focuses on patient and hospital characteristics related to pneumonia mortality in the United States and analyzes how health disparities are influenced by geographical location and socioeconomics.

**Implications for the public**

Pneumonia is a severe multifaceted disease affected by patient and hospital factors. Despite available treatment, patients continue to die from this disease in the United States and beyond. Understanding risk factors involved with in-hospital mortality can improve patient care and provider understanding of the community they serve. Implications from our study include promoting preventative strategies and assistance programs for disadvantaged patients. Hospitals serving low-income or rural patients may see worse health outcomes and require greater resource allocation. Regions like the East South Central, which had one of the highest mortality rates, significant poverty rates, and many rural areas could benefit from such assistance programs

Pneumonia is one of the leading causes of hospital admission in the United States,^1^ with a global health burden of about 6.8 million hospitalizations and 1.1 million deaths in patients over 65 years old in 2015.^2^ Pneumonia is a lung infection caused by either fungi, bacteria, or viruses, leading to moderate to severe disease characterized by cough, fever, and trouble breathing.^3^ In the United States, it mainly affects adults, with about 1.5 million people presenting to the Emergency Department with pneumonia in 2018,^3,4^ causing about 40 000 pneumonia-related deaths.^3^ Pneumonia is preventable through the pneumococcal vaccine^4^ and is treatable with antibiotics or antivirals.^3^ However, in 2020, only about 25% of people in the United States over 18 years old had received the pneumococcal vaccine.^4^

 There are three main ways to classify pneumonia based on how it is acquired, in the community, in the hospital, or through healthcare.^5^ Community-acquired pneumonia is the leading cause of infectious disease death and is correlated with increased risk for in-hospital mortality.^5,6^ Factors associated with in-hospital mortality include comorbidities like chronic heart and respiratory disease and age (also associated with increased severity and comorbidities), which hospitals cannot control.^5,6^ Other community-acquired pneumonia risk factors include diabetes, alcohol intake, HIV, chronic renal failure, and leukemia or lymphoma.^7^ Similarly, ventilated hospital-acquired bacterial pneumonia and non-ventilated hospital-acquired pneumonia increasingly affect older patients with more comorbidities.^8^ However, non-ventilated and ventilated hospital-acquired pneumonia had higher hospital charges, length of stay, and mortality rates than patients with community-acquired pneumonia.^9^ One study contends that because non-ventilator-associated hospital-acquired pneumonia is associated with high mortality rates but generally unclear risk factors, all patients should be monitored, and strong prevention efforts should be promoted.^10^

 Understanding patient and hospital factors that increase pneumonia mortality rates outside of common clinical characteristics is necessary. For example, patient demographics like low socioeconomic status and race are associated with worse disease outcomes.^11,12^ There is also some discussion on how the geographic location of hospitals may affect patient outcomes based on surrounding community demographics.^11-16^ It is now vital to connect patient and hospital factors to better characterize differences in patient needs in various locations and address possible health disparities in pneumonia outcomes.

 Socioeconomic status and race have important implications on pneumonia outcomes. One study discussed how systemic race and socioeconomic factors play more significant roles in patient outcomes than hospital factors.^17^ Generally, patients with low socioeconomic status tend to have an increased risk of in-hospital mortality, decreased palliative care usage, worse health outcomes, decreased life expectancy, and earlier disease onset.^18,19^ Further, differences in insurance have also impacted health outcomes, with Medicaid and Medicare patients having worse results than private insurance and Medicaid patients faring the worst.^18,19^ Lower reimbursement rates than private insurance may be to blame.^18,19^

 Differences also tend to appear in the outpatient setting. Those with lower socioeconomic factors have higher one-year mortality rates, an increased risk for respiratory infections and community-acquired pneumonia, and greater disease severity.^20^ Hospitalized pneumonia patients, in general, have high 1-year mortality rates, possibly due to chronic inflammation exacerbating comorbidities.^20^ Minorities are at increased risk for comorbidities with higher disease rates and lower socioeconomic status and health outcomes.^11,12,21^ Minority hospitals penalized for performance outcomes face more significant economic challenges as their patients may already have decreased healthcare access due to low socioeconomic status.^12^

 Another study outlined the demographics of children with pneumonia. They found higher incidence rates in the South; more than half of the patients had Medicare, and care was more expensive in the West.^22^ Further, there are similar mortality rates between large and small hospitals, and greater severity/morality could be related to socioeconomic status.^22^ For different disease states, results are mixed on if hospital teaching status affects disease outcomes,^23,24^ and such a connection has yet to be shown for pneumonia. Previous research on health outcomes from teaching vs. non-teaching hospitals has found that public safety-net hospitals, treating patients regardless of insurance, tend to have worse results, treat more severe patients, and can still be costly, possibly because of patient sociodemographic factors.^24-26^

 Other hospital factors that may be related to pneumonia outcomes include geographic location. Few studies have connected the geographic location of hospitals to pneumonia mortality. However, community demographics and geographic location can affect hospital quality. Unfortunately, hospitals in underserved regions may not have sufficient resources, leading to poor quality scores, influencing coverage decisions, and creating financial obstacles exacerbating the situation.^16^ Previous research describes how minority-serving hospitals have been unable to decrease vital disease outcomes like mortality and length of stay compared to non-minority-serving hospitals.^12^ One study looked at flu and pneumonia rates for hospitalized long-term care facility patients by geographic location between 2015-2016. Bosco et al found that the Midwest and the Southern US had the highest incidence rates and risk-standardized incidence rates.^14^ Differences in hospitalizations may have varied by location based on staffing, vaccination rates, and hospital relations to the care facilities.^14^

 There is a lack of research focusing on how patient factors, like race and socioeconomic demographics, and hospital factors, like location, size, and teaching status, are related to pneumonia death. Though there have been discussions on these characteristics separately or towards readmission rates, there is a need to identify their impact on pneumonia mortality rates. With pneumonia being a common preventable and treatable disease, it is vital to determine the factors driving mortality rates outside of treatment and vaccination rates. This study will explore patient and hospital characteristics related to pneumonia mortality in the United States and analyze how health disparities are influenced by geographic location and socioeconomics. Such descriptions may pinpoint necessary changes to public health programs and promote hospitals re-evaluating their compliance with standards of care for pneumonia and any obstacles to doing so.

## Materials and Methods

###  Data Collection

 The latest 2016-2019 National Inpatient Sample (NIS) database was used to obtain a population-based estimate for nationwide patients with pneumonia. Among all 2016-2019 NIS samples (N = 28 484 087), shown in Figure, we first identified a primary diagnosis of pneumonia (J189, n = 394 455) using the International Classification of Diseases 10th Revision (ICD-10-CM/PCS) codes for pneumonia. Then, after patients with missing variables were excluded, we obtained patients with pneumonia for final analysis (n = 374 766, weighted n = 1 873 828). We collected our samples from the NIS. Although we used NIS data for the analysis, our collected samples from the NIS are independent of the NIS.

**Figure:**
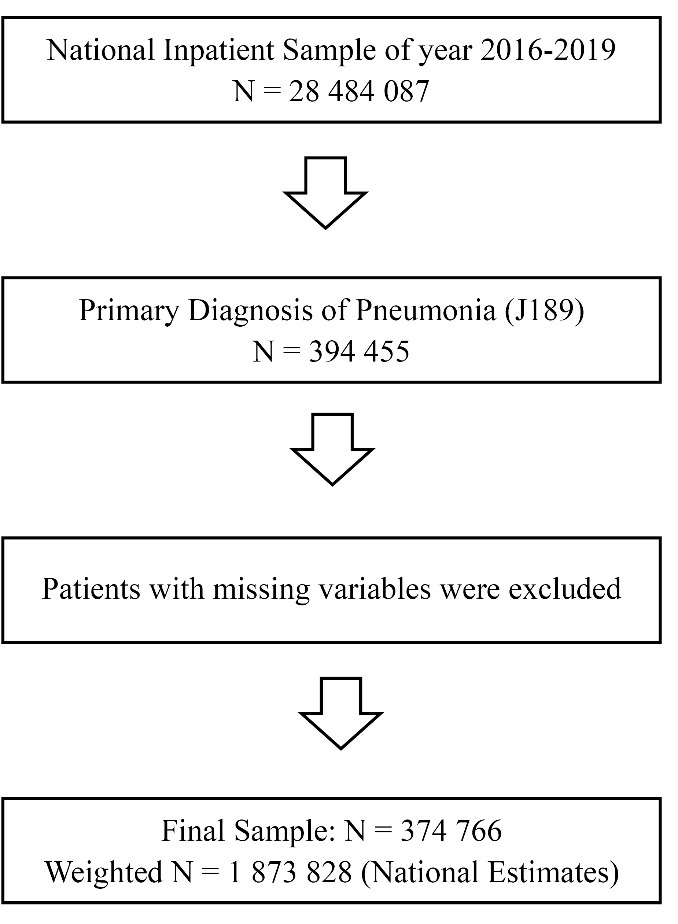
Flow Chart of Sample Selection.

###  Variables

 The primary outcome of this study was to investigate the factors associated with in-hospital death among pneumonia patients. Therefore, the dataset included the “Died during hospitalization” variable. In addition, we adjusted for various patient and hospital confounders. Patient characteristics included age, race, annual median household income, primary payer (Medicare, Medicaid, Self-Pay/No Charge, Other, and Private insurance), and the severity of illness. Hospital characteristics include bed size, ownership, location, teaching status, and region.

###  Statistical Analysis

 Sampling weights were applied to all statistical analyses to represent nationwide pneumonia patients. First, we examined the characteristics of the final dataset, which included patient/hospital characteristics by in-hospital death status. Patient/hospital characteristics were presented as weighted frequency (percentage) or means (standard deviation). Rao-Scott chi-square tests were used for categorical variables to investigate groups. Then we explored how patient and hospital characteristics were associated with in-hospital death using multivariate survey logistic regression analysis. Finally, we conducted an investigation where we ran the model with different regional variables. The additional analysis was performed by adjusting all other variables. All studies used SAS statistical software (version 9.4; SAS Institute Inc., Cary, NC, USA). All statistical tests were two-sided, and statistical significance was determined at *P* < .05.

## Results

###  Patient/Hospital Characteristics and Descriptive Statistics

 A total of 374 766 pneumonia patients were identified in the 2016-2019 NIS data (weighted n=1 873 828, Table 1). Among them, 8479 (weighted n = 42 395, 2.26%) died during hospitalization. The general characteristics of patients and hospitals are presented in Table 1.

**Table 1:** General Characteristics of Sample

	**In-Hospital Death**				***P *** **Value**
	**Yes**		**No**		
	**No.**	**%**	**No.**	%	
N	8479	2.26	366 287	97.74	
Weighted N [National Estimates]	42 395	2.26	1 831 433	97.74	
Age^a^	64.24	23.08	77.03	12.95	<.0001
Gender					.0002
Male	20 715	2.36	857 704	97.64	
Female	21 680	2.18	973 729	97.82	
Race					<.0001
White	32 900	2.43	1 320 863	97.57	
Black	4170	1.73	236 510	98.27	
Hispanic	3115	1.77	172 935	98.23	
Asian or Pacific Islander	970	2.33	40 660	97.67	
Native American	185	1.41	12 950	98.59	
Other	1055	2.17	47 515	97.83	
Median household income					0.0764
0-25th percentile	14 035	2.23	614 284	97.77	
26th to 50th percentile	11 675	2.27	503 609	97.73	
51st to 75th percentile	9135	2.20	405 820	97.80	
76th to 100th percentile	7550	2.39	307 720	97.61	
Primary payer					<.0001
Medicare	32 670	2.73	1 165 949	97.27	
Medicaid	2180	0.84	257 875	99.16	
Private insurance	5300	1.66	314 655	98.34	
Self-pay	585	1.06	54 695	98.94	
No charge	50	1.18	4170	98.82	
Other	1610	4.51	34 090	95.49	
Severity of Illness					
No/Minor comorbidity or complications	595	0.29	204 180	99.71	<.0001
Moderate comorbidity or complications	3645	0.54	671 344	99.46	
Major comorbidity or complications	14 860	1.94	752 814	98.06	
Extreme comorbidity or complications	23 295	10.29	203 095	89.71	
Bed size of the hospital					<.0001
Small	10 815	2.11	502 129	97.89	
Medium	12 720	2.24	555 224	97.76	
Large	18 860	2.38	774 080	97.62	
Ownership of hospital					0.0006
Government nonfederal	5725	2.34	238 593	97.66	
Private not-profit	30 670	2.29	1 305 745	97.71	
Private invest-own	6000	2.05	287 095	97.95	
Location/teaching status of the hospital					.0185
Rural	8130	2.40	330 399	97.60	
Urban nonteaching	11 275	2.27	486 454	97.73	
Urban teaching	22 990	2.22	1 014 580	97.78	
Region of hospital					<.0001
Northeast	8220	2.42	332 020	97.58	
Midwest	8940	2.09	419 499	97.91	
South	17 610	2.20	784 504	97.80	
West	7625	2.52	295 409	97.48	

^a^Mean/standard deviation.

Table 2 shows temporal trends of in-hospital death of nationwide pneumonia patients during 2016-2019. Again, we found a decreasing in-hospital death rate among national pneumonia patients over the years (2.45% in 2016, 2.32% in 2017, 2.25% in 2018, and 2.19% in 2019).

**Table 2:** Temporal Trend of In-Hospital Death of Pneumonia Patients

	**2016**	**2017**	**2018**	**2019**
N	113 417	87 780	96 646	76 923
Weighted N [National Estimates]	567 084	438 900	483 229	384 615
In-hospital death				
No	553 504	428 965	472 595	376 370
Yes	13 580	9 935	10 635	8245
% Of yes	2.45%	2.32%	2.25%	2.19%

###  Factors Associated With In-Hospital Death 

 The factors associated with in-hospital death from the survey logistic regression model are shown in Table 3. After controlling for all other variables, patient characteristics include older age, male, 0-25th percentile household income, payer other than Medicare, and severe comorbidity or complications were associated with higher odds of in-hospital death. Furthermore, hospital characteristics such as government or rural status and location in the Northeast, South, or West were associated with higher in-hospital death odds than their reference groups.

**Table 3:** Results of Survey Logistic Regression Models: Factors Associated With In-Hospital Death

**Variables**	**OR**	**95% CLs**	
Age	1.046	1.043	1.048
Gender			
Male	1.103	1.055	1.154
Female	1.000		
Race			
White	1.000		
Black	1.033	0.956	1.116
Hispanic	1.017	0.931	1.112
Asian or Pacific Islander	1.038	0.891	1.208
Native American	0.904	0.650	1.258
Other	1.178	1.019	1.361
Median household income			
0-25th percentile	1.102	1.025	1.184
26th to 50th percentile	1.027	0.958	1.102
51st to 75th percentile	0.964	0.897	1.036
76th to 100th percentile	1.000		
Primary payer			
Medicare	1.000		
Medicaid	1.294	1.160	1.444
Private insurance	1.660	1.540	1.790
Self-pay	1.578	1.302	1.914
No charge	1.668	0.886	3.139
Other	3.063	2.690	3.489
Severity of illness			
No/Minor comorbidity or complications	1.000		
Moderate comorbidity or complications	1.311	1.079	1.592
Major comorbidity or complications	4.539	3.776	5.457
Extreme comorbidity or complications	28.267	23.529	33.960
Bed size of the hospital			
Small	1.014	0.958	1.073
Medium	0.973	0.922	1.026
Large	1.000		
Ownership of hospital			
Government, nonfederal	1.000		
Private, not-profit	0.828	0.773	0.887
Private, invest-own	0.748	0.686	0.815
Location/teaching status of the hospital			
Rural	1.307	1.225	1.394
Urban nonteaching	1.012	0.959	1.068
Urban teaching	1.000		
Region of hospital			
Northeast	1.285	1.196	1.380
Midwest	1.000		
South	1.137	1.068	1.210
West	1.202	1.115	1.295
Year	0.845	0.827	0.863

Abbreviations: OR, odds ratio; CLs, confidence limits.

Table 4 shows the primary model results with more specific region variables. We used nine categories for census division, and the reference group is East North-Central. Except for the Mountain region, every division has higher in-hospital death odds than East North-Central.

**Table 4:** Results of Survey Logistic Regression Models Using Census Division of Hospital

**Variables **	**OR**	**95% CLs**	
Census division of hospital			
New England	1.288	1.148	1.446
Middle Atlantic	1.373	1.262	1.493
East North Central	1.000		
West North Central	1.189	1.070	1.321
South Atlantic	1.123	1.038	1.215
East South Central	1.335	1.215	1.467
West South Central	1.214	1.107	1.330
Mountain	1.019	0.907	1.146
Pacific	1.399	1.279	1.530

Abbreviations: OR, odds ratio; CLs, confidence limits. * All other variable were adjusted.

## Discussion

 The current study has demonstrated that in-hospital pneumonia mortality rates may be affected by geographic region, insurance status, hospital ownership, and urban vs. rural location. These hospital and sociodemographic factors have vital implications for health disparities. Like previous research, we found increased mortality rates amongst elderly patients and patients with severe comorbidity or complications.^5,6,8,9^ Unlike studies on other disease states,^18,19^ which found that Medicare was associated with higher mortality, we had a higher odds ratio for those with private insurance. Looking at hospital-related factors, we found higher mortality rates for rural hospitals than urban and more significant mortality rates across the Northeast, South, and West.

 Socioeconomic differences had a small but significant effect on mortality rates. The lack of a substantial impact could be because hospital treatments are largely standardized regardless of socioeconomic status.^17,20^ The current study found that patients in the 0-25th percentile for median household income had slightly higher in-hospital mortality rates. Previous research characterized how hospitals treating patients in low socioeconomic areas may have poorer outcomes because patients present with more severe disease^12^ partly due to differences in the built environment.^27^

 With wealth disparities across the United States, it becomes necessary to identify the geographic location of hospitals with increased pneumonia mortality to discuss the expanding needs of low socioeconomic populations. In 2019, 34 million people lived in poverty^28^; thus, patients’ socioeconomic status remains a vital health determinant. Our study found higher in-hospital mortality rates in the South, Northeast, and West. Looking into more specific regions, we found the highest mortality rates in areas like the Pacific, East South Central, and Middle Atlantic.

 The Pacific had 9.5%-11.9% of their population living in poverty in 2019.^29^ East South Central, with the second-highest in-hospital mortality, had 12%-15% and higher of their people in poverty.^29^ The Middle Atlantic had about 9.2%-13% of their population living in poverty in 2019.^29^ Wealth may affect health and has been related to worse health outcomes for other disease states.^18,19^ According to Healthy People 2030, economic factors are part of the social determinants of health as they can affect access to care, healthy food, and housing.^30^

 Private insurance and self-pay had higher in-hospital pneumonia mortality rates than Medicare and Medicaid patients. Our finding differs from previous research, which found that Medicare and Medicaid patients typically have worse health outcomes.^18,19^ Because treatments are standardized,^17,20^ insurance reimbursement rates may not have had less impact. Lack of insurance for self-pay patients may also have affected access to healthcare.

 Another contributing factor may be hospital status. Areas like the East South Central, South Atlantic, New England, and West North Central had some of the highest mortality rates, possibly due to rural areas.^31^ Rural hospitals may have an increased risk for poor health outcomes.^32^ Mortality rates for rural hospitals have not improved significantly as urban hospitals and rural residents have a decreased life expectancy.^32^ Rural patients may have worse water quality, less education, increased preventable death, and more significant socioeconomic barriers to health.^32^ For example, previous research found that in-hospital mortality rates for myocardial infarctions were raised in rural hospitals compared to urban teaching or non-teaching.^33^ Similarly, the current study saw rural hospitals had higher in-hospital pneumonia mortality rates than urban teaching or non-teaching.

 The ownership of the hospital was separated into government-owned, private not-for-profit, and private investor-owned. Our study found lower hospitality mortality rates for private not-for-profit and investor-owned than government-owned. Studies on other disease states also found that government hospitals have worse health outcomes than private investor-owned hospitals.^34^ Our findings suggest that hospitals may need to re-evaluate their compliance with existing pneumonia care standards and identify barriers to achieving compliance. In addition, small, rural, and government hospitals may need further discussion on improving outpatient comorbidity management and education to mitigate disease complexity and examine their existing care for pneumonia patients.

 This study has described the geographic and patient factors that may influence in-hospital pneumonia outcomes, highlighting minority health disparities across the United States; however, there are limitations to this research. First, the data set was from 2016 to 2019, which may reflect something other than the current situation. With the COVID-19 pandemic, pneumonia rates have increased globally. Secondly, the National Inpatient Dataset uses International Classification of Diseases-10th Version-Clinical Modification/Procedure Coding System (ICD-10-CM/PCS) codes for pneumonia which could have restricted patient selection. Approximately 5% of data were excluded due to missing race or insurance status information to achieve a more uniform dataset. This method is consistent with previous research using NIS data.^35-37^ Still, excluding records may have impacted the study’s findings. Another area for improvement is the lack of clinical information and severity in the dataset, which may impact the real-life implications of our results. The NIS dataset does not include information on patient transfers between hospitals and intensive care unit vs. non-intensive care unit mortality rates, which could affect overall mortality rates between hospital types.

 Additionally, because of limitations with the dataset, we cannot gauge compliance to guideline therapy based on hospital resources, such as access to a pulmonologist in small hospitals or standard treatment for community-acquired pneumonia. Despite these limitations, the current study described multiple patient and hospital factors across the United States, making it generalizable. The results described here investigate health disparities and identify areas where public health programs may significantly impact patient outcomes by promoting vaccinations, chronic disease management, and education on respiratory infections.

## Conclusion

 Pneumonia is a severe multifaceted disease affected by patient and hospital factors. Despite available treatment, patients continue to die from this disease in the United States and beyond. Understanding risk factors involved with in-hospital mortality can improve patient care and provider understanding of the community they serve. Our analysis found that in-hospital pneumonia rates are related to older age, increased disease severity and comorbidities, income, insurance type, hospital location (rural or urban), ownership, and census region. The connections to older age, disease severity, and comorbidities are well studied in the literature. However, our exploration of socioeconomics and rural location has strong connotations. Poverty and rural areas played heavy roles in pneumonia mortality and could translate to other disease states. Healthy People 2030 has outlined the need to overcome economic barriers to health; more outstanding research is required to understand how assistive programs impact patient care and best address health disparities in rural and low-income areas. Implications from our study include promoting outpatient chronic disease management and prevention to reduce mortality risk and disease complications. Greater vaccination education and rates could also help prevent disease rates and severity. Public health programs should discuss the different types of respiratory infections and when individuals should seek medical treatment. Regions like the East South Central had one of the highest mortality and significant poverty rates, and many rural areas could benefit from such public health programs.

## Ethical issues

 The data we use was secondary data and all patient data were encrypted and unable to identify. This study was approved for waiver from the Institutional Review Board of Soonchunhyang University (202203-SB-027).

## Competing interests

 Authors declare that they have no competing interests.
